# Shared Situational Awareness within the Hospital Emergency Context: A Scoping Review

**DOI:** 10.3390/healthcare10081542

**Published:** 2022-08-15

**Authors:** Modi Al-Moteri, Abeer Abdulaziz Alfuraydi, Aliya Z. Alsawat, Riyadh Saleh Almulhis, Bashaer Salem Alnadwi, Hanan A. M. Youssef, Ensherah Saeed Althobiti

**Affiliations:** 1 Nursing Department, College of Applied Medical Sciences, Taif University, Taif 21944, Saudi Arabia; 2 Emergency Department, King Salman Bin-Abdulaziz Medical City, MOH, Madinah 2480, Saudi Arabia; 3 Emergency Department, Security Forces Hospital, MOI, Makah 11481, Saudi Arabia; 4 King Abdulaziz Specialist Hospital, Ministry of Health, Taif 21944, Saudi Arabia

**Keywords:** shared, situational awareness, emergency, hospital

## Abstract

**Background.** Shared Situation Awareness (SSA) has been applied in many fields such as sport, the military and aviation with promising outcomes on team performance. The application of SSA within the hospital emergency healthcare context has not been explored yet. The aim of this scoping review is to explore and map literature related to shared situational awareness within the hospital emergency healthcare context. **Methods.** The Arksey and O’Malley (2005) framework was used in which three electronic databases were searched for evidence investigating SSA within a hospital emergency healthcare context. **Results.** A review of the literature showed a clear lack of evidence that directly investigates SSA within the context of hospital emergency care. In the emergency medical field, the term SSA is seldom used and ‘team situation awareness’ is the most frequently used term. The most common framework was the three-level framework. Two techniques were reported in the selected studies to investigate SSA (1) freeze probe technique and (2) observer-based rating technique. The freeze probe technique mandates a simulation or artificial environment, while the observer-based rating technique could be applied in an ecological as well as an artificial environment. There is no standardized technique to calculate the score of the SSA. Finally, there was a significant impact of SSA on clinical team performance as well as some related skills such as leadership, task management, mindfulness and task prioritization. **Conclusions.** Reviewing the literature revealed a lack of studies investigating the use of SSA within the context of hospital emergency care. There is also a lack of agreement on how a SSA score should be calculated. Further studies are required to overcome these issues.

## 1. Introduction

Situational awareness (SA) refers to an awareness of the information surrounding a particular situation and the understanding of this information in order to anticipate what might happen in the near future [1,2]. In the simplest terms, SA allows individuals to keep an eye on the “big picture” of a situation while managing details and issues as they evolve. While the health literature shows that SA is important for the performance of an individual healthcare provider [3], there is no agreement on team SA and its relationship to the performance of the team. In cognitive psychology literature, “team SA” is known as shared situational awareness (SSA) [4]. SSA impact on team performance is well documented in sport [5], aviation [6] and the military [7].

In the context of emergency medicine, as in many other contexts, the necessity for SSA becomes heightened by the presence of emergency team members. A team in the medical emergency context consists of a number of healthcare providers working collaboratively, interdependently and adaptively toward a specific goal for responding to a patient’s life-threatening situation [8]. There are two types of team SA: (1) complementary SA, in which the team members have SA that does not overlap but is complementary, resulting in the needed team SA; and (2) shared SA, in which team members share the same SA [4] (p. 100). For instance, in a cardiopulmonary resuscitation (CPR) team, the team mainly consists of nurses, physicians and respiratory therapists of which each will have their own roles and for which they have their own SA. However, all of them need to function on shared information and the actions of one can have an impact on the actions of the others. The collaborative interdependent function of team members requires SSA which acts as a foundation to effective team performance. 

SSA has been applied in many fields with promising outcomes on team performance [4]. However, the application of SSA within the hospital emergency healthcare context has not yet been explored. To address this gap in knowledge, this scoping review will explore and map the literature related to shared situational awareness within the hospital emergency healthcare context. 

## 2. Materials and Methods

The Arksey and O’Malley [9] framework was used in the current study to guide the search process for primary evidence aimed to explore the skills of shared situational awareness in the medical emergency situation. The framework is known to be appropriate when the area of investigation has not been well-addressed yet [10]. The Arksey and O’Malley [9] framework includes five steps as follows:

- Identifying the research question,
- Identifying relevant studies,
- Selection of eligible studies,
- Charting the data,
- Collating and summarizing the results.

### 2.1. Identifying the Research Question

There are few studies that specifically and directly investigate shared situational awareness (SSA) in a medical emergency situation when compared to the volume of SSA studies in other disciplines such as the military, aviation and sport. This scoping review aimed to search the literature for the available evidence related to the following research questions:

1. What are the different approaches categorized as SSA within the hospital emergency healthcare context?
2. What are the techniques used to assess SSA within the hospital emergency healthcare context?
3. What are the techniques used to calculate SSA within the hospital emergency healthcare context?
4. What are the impacts of SSA that have been clearly linked to team performance and related skills within the hospital emergency healthcare context?

### 2.2. Identifying Relevant Studies

To identify the relevant studies, specific search strategies were applied. The search process targeted studies that (1) focused on inpatient medical emergency context in relation to shared situation awareness, (2) were published in English after 2012 and (3) have been peer reviewed. Studies were excluded if they (1) did not contain the phrase “shared situation awareness” or “team situation awareness”, or (2) did not pertain to a medical emergency context. Three databases—PubMed, Science Direct and ProQuest—were searched, as well as Google Scholar. The following “search keywords” were used to search for relevant literature: “emergency”, “medical team”, “shared”, “healthcare”, “awareness”, “situation *”, “situational awareness”, “member”, “nurs *”, “physician”, “CPR”, “Code blue”, “emergency care”, “decision-making”, “Communicat *”.

### 2.3. Selecting of Eligible Studies

Initially, the search outcomes revealed large number of irrelevant studies. When the selection criteria for this review were restricted to “shared situational awareness” in hospital medical emergency context, many of the retrieved studies were investigating situation awareness for individuals. Therefore, the study settled on a combination of concise primary search terms— “emergency” AND “medic *” AND “team” OR “shared” AND “awareness” AND “situation *”.

### 2.4. Data Charting

Guided by the review questions, researchers have extracted data independently. Then, the extracted data were discussed and summarized in a preformed form. The form included study title, date of publication, study aim and design, SSA approach, techniques used to assess SSA, calculating SSA score, and team performance and related skills linked to SSA. Although assessing the quality of the selected evidence is a preferred step for most reviews that involve a systematic and comprehensive search, scoping reviews do not require that. Skipping the methodological quality assessment is what distinguishes a scoping review from other types of reviews [10]. A scoping review is exploratory in nature and explores less established areas of investigation Table 1.

### 2.5. Summarising the Results 

**Figure 1:**
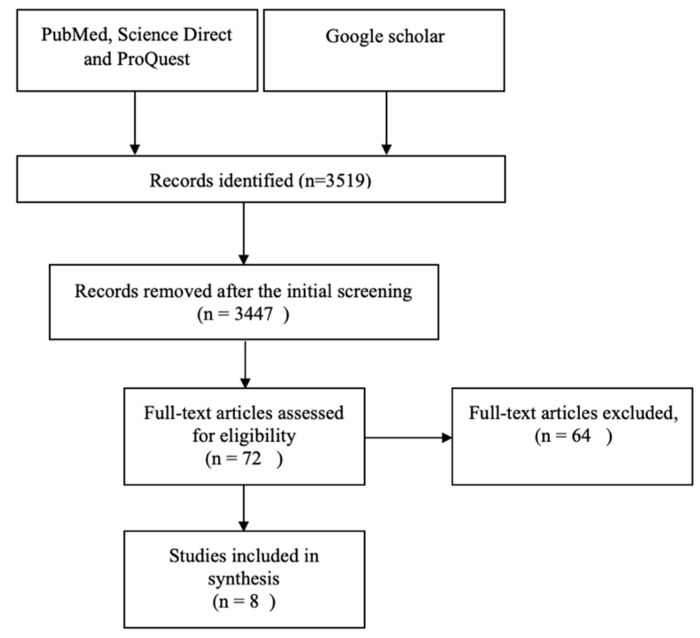
Flow diagram for literature search and screening.

Initial searching of the electronic database yielded 3519 studies. After removing reports, reviews, documentaries, theses, duplications and screening the title and the abstract, 3447 were excluded. The full-text of the 72 articles were screened to identify eligibility. Of these, 30 studies were excluded based on the absence of clear measurement of situational awareness, 23 studies were from an irrelevant context (mainly from emergency management system), six were excluded based on the absence the full text (conference abstract); in three studies, the full text was only available in a non-English language, one study was not peer reviewed and one was an RCT protocol. A total of eight study findings were collated and analyzed Figure 1.

## 3. Results

Eight studies were included in the analysis of the current review. Seven of these studies employed an observational design [11,12,13,14,15,16,17]. One was a randomized controlled intervention [18]. The results of this review showed that the term “shared situational awareness” in itself was not commonly used; instead “team situational awareness” was the most commonly used term [1,2,3,4,5,6,7,8,9,10,11,12,13,14]. Further, the term “shared information” and shared mental model were used interchangeably with respect to team situational awareness [15,17]. “Team non-technical skills” was also used to describe situational awareness of each team member [16].

### 3.1. Shared Situation Awareness Frameworks in Medical Emergency Context

Although, SSA is not globally used in the hospital emergency context [19], the review results revealed that Endsley’s frameworks [2] were the most used SA frameworks in the hospital emergency context. Four studies [11,12,15,18] employed the three-levels of Endsley’s framework [2], while one study has used one level of the framework [13]. According to Endsley’s framework [2], situational awareness components are Level 1: perception of the information present in the situation, Level 2: comprehension of what the information present in the situation means and, Level 3: projection of what might happen in the situation in the near future. One study developed its own team resuscitation SA framework that included seven dimensions [14], and another two studies set its observable behavior indicators [16,17]. 

### 3.2. Techniques Used to Assess Shared Situational Awareness

This review showed that Situation Awareness Global Assessment Technique (SAGAT) was the most common used technique [11,12,13,15,18]. SAGAT is a global technique designed by Endsley [1] to measure situational awareness. The technique provides an objective and direct measurement of situational awareness [2]. O’Neill et al. [14], Krage et al. [16] and Johnsen et al. [17] have used behavioral markers. In subsequent paragraphs, the elements required to apply SAGAT will be discussed meticulously. 

#### 3.2.1. Simulation

For optimum utilization of SAGAT, simulation need to be employed. Various medical emergency simulated scenarios were developed such as respiratory distress progressing to cardiac arrest [13], trauma, cardiac and shock resuscitation [15], obstructive pulmonary disease and severe septic shock [18], two types of trauma (adult pedestrian hit by a car; and construction worker who has fallen 6 m from a scaffold) [17], neurotrauma, deteriorating case of ischemic bowel obstruction (volvulus), anaphylactic reaction to a blood transfusion [12], ventricular fibrillation, pulseless ventricular tachycardia [16] and hypovolemic shock complicated by cardiac arrest [14].

**Table 1:** Included studies characteristics.

Author, Year	Study Aim	Design	Main Findings
Rosenman ED., 2018 [13]	To develop and evaluate a novel approach to simulation-based TSA measurement in interprofessional EM teams.	Observational Study	Three-levels of Endsley’s (1995) framework (Perception, comprehension, projection), however, only projection was investigated. Situation Awareness Global Assessment Technique (SAGAT), simulation, probe questions. The collective agreement among team members answers were analyzed. There was a significant relationship between SSA and team clinical performance.
O’Neill TA., 2018 [14]	To identify a multidimensional taxonomy that captures the complexity of team SA in behavioral terms	Pre-post-observational design	Behavioral markers, trained observers. Video-recorded simulated resuscitation. Observation based rating. The mean of the raters scores of SSA was identified. No association between team performance and SSA was initiated.
Parush A., 2017 [15]	This study focused on the design of an ED situation display and pilot test its influence on teamwork and situational awareness during simulated resuscitation scenarios.	Observational study	Three-levels of Endsley’s (1995) framework (Perception, comprehension, projection). Situational Awareness Global Assessment Technique (SAGAT) tool, freezing activities, simulation approach, probe questions. Calculating the mean of the SA scores. Significant relationship between SAA and team clinical performance.
Jonsson K., 2021 [18]	To evaluate an educational program on situation awareness for interprofessional teams at the intensive care units using team and task performance as outcomes.	Randomized Controlled Intervention Study	Three-levels of Endsley’s (1995) framework (Perception, comprehension, projection). Situation Awareness Global Assessment Technique (SAGAT), simulation, and probe question. Calculating the mean of the SA scores. Leadership and task management skills has improved.
Coolen E., 2019 [12]	To measure SA among team members during simulation of acute pediatric care scenarios on the medical ward and its relationship with team effectiveness	Observational design	Three-levels of Endsley’s (1995) framework (Perception, comprehension, projection). Situation Awareness Global Assessment Technique (SAGAT), simulation, probe questions. The collective agreement among team members answers were analyzed. Improvement in leadership and team task management skills when situational awareness was enhanced.
Chmielewski J., 2021 [11]	To investigate whether mindfulness is related to the SA levels among final-year medical students confronted with life-threatening situations using direct and objective methods, and a simulation freeze technique	Observational design	Three-levels of Endsley’s (1995) framework (Perception, comprehension, projection). Situation Awareness Global Assessment Technique (SAGAT), simulation, freezing probe. The mean scores pf SA was calculated to represent SSA. No relationships were found between other components of mindfulness and students’ SA during the simulation.
Krage R., 2017 [16]	To investigate the relationship between nontechnical and technical skills under control conditions and when external stressors are present.	simulator-based randomized cross-over study	Simulation approach. Video recordings of the CPR scenarios. Non-technical performance was scored using the Anaesthetists’ Non-Technical Skills (ANTS) score. Technical performance was assessed by pre-developed technical skills score. A significant correlation between non-technical and technical performance scores was observed when external stressors were present.
Johnsen BH., 2017 [17]	Based on the theoretical framework of shared mental models (SMM): (1) To investigate whether behavioral markers of SMM in team leaders are associated with subject matter expert (SME) rating of team performance. (2) To investigate whether the higher performing teams were characterized by more frequent behavioral markers of SMM.	Observational Study	Behavioral markers of a shared mental model. Video recordings trauma-team leaders training in simulated emergency situations. Anti-Air Teamwork Observation Measure (ATOM). No difference was found for the behavioral marker of team initiative, measured as bringing up suggestions to other team members.

#### 3.2.2. Freezing Activity

Simulation allows the freezing of the scenario at a predetermined time while participants quickly answer questions related to the three levels of situation awareness (perception, comprehension, and projection). Four studies employed freezing activity [11,12,15,18]. Rosenman et al. [13] only assessed the projection level of situational awareness, hence, no freeze technique was used, and the situational awareness questionnaire score was collected at the end of the simulated scenario. O’Neill et al. [14], Krage et al. [16] and Johnsen et al. [17], on the other hand, used an observable behaviors sheet scored by trained observers. 

#### 3.2.3. Probe Questions 

Five studies employed probe questions to assess the three levels of situational awareness [11,12,15,18]. Rosenman and colleagues [13], however, assess only the projection level of situational awareness. Rosenman and colleagues [13] claimed that projecting is assumed to be most important element for team performance in highly dynamic environments. O’Neill et al. [14], Krage et al. [16] and Johnsen et al. [17] did not use probe questions however and instead they relied on raters who were trained in the use of the SA assessment tool for the observable behaviors. 

### 3.3. Calculating Situational Awareness Score 

Various techniques were reported in the selected studies to calculate the score of the SSA. Some studies have collected complementary situational awareness scores from team members, then, SSA was collated by analyzing the collective agreement among team members answers [12,13]. Other studies measured only complementary situational awareness and the mean of their scores was calculated to represent team situational awareness [11,15,18]. As O’Neill et al. [14], Johnsen et al. [17] and Krage et al. [16] have not used freeze-probe activity, they calculated the mean of the raters scores of SSA.

### 3.4. Effect of SSA on Team Performance and Other Related Skills 

The selected studies reported a significant impact of SSA on team performance and some related skills. Some studies reported a significant relationship between SSA and team clinical performance [13,15,16,17]. Jonsson et al. [18] reported improvement in leadership and team task management skills when situational awareness was enhanced. Coolen et al. [12] noticed a strong correlation between SSA and task prioritization. Chmielewski et al. [11] reported a positive relationship between Level 1 of situational awareness and overall mindfulness score.

## 4. Discussion

Measuring SSA or Team SA is critical aspect of assessing the effectiveness of team performance. The current scoping review investigated SSA within the hospital emergency healthcare context. Two techniques were reported in the selected studies to investigate SSA: (1) freeze probe technique (SAGAT) and (2) observer-based rating technique. As demonstrated in this review SAGAT [1] is the most commonly tool used to assess both complementary SA and SSA. However, SAGAT in its current form is reliable only when assessing complementary SA. To overcome such limitation, SAGAT queries must be designed in a way that include assessment of the common complementary SA of each team member and the specific SSA requirements. Then, responses to queries related to the particular SSA can be compared to identify if the same responses are made across team members. This type of measurement can identify the cause of breakdowns in team performance. On the other hand, “observer-based rating technique” uses trained observers to rate specific “observable actions” that are mainly related to the task at hand while incorporating some of SA components. However, there is a great debate around the reliability of the technique in assessing SSA [4]. Several issues were identified [4]: (1) the observers must have high level of domain specific knowledge in order to appropriately perform the observation; (2) it requires more than one observer and inter-observer agreement must be established prior; and (3) there is a focus on observable action rather than underlining cognition processes. What is really missed here is a body of knowledge that better investigates the SAGAT probe technique and observer-based rating technique in relation to SSA. 

Additionally, it is important to mention here the methodological limitation of the application of the SAGAT probe technique and observer-based rating technique in the medical emergency field where studies are mainly conducted in an artificial environment. Although lab-based investigation is critical for building a foundation for the area of cognitive and human factor research, it is less reliable than investigation in ecological environments. Indeed, human behavior may differ between lab-based techniques and their more “real-world” situation. It is perhaps recommended for medical emergency researchers to study SSA in real emergency situations. 

SSA has been comprehensively investigated in other fields such as aviation and the military and was able to provide insights into how it might be linked to team performance [20,21]. Given the scarcity of studies retrieved in this review, it is hard to make any claims in linking SSA directly to team performance within the context of hospital emergency. Conducting more studies would benefit the field in which one might be able to identify the relationship between SSA and team performance. Hence, it would be argued that this be considered an appropriate area for investigation. Although assessing complementary SA is somewhat well established in literature [22,23,24], more research is required in SSA area and the research evidence must be connected to the team performance. Indeed, it was noted that teams may suffer from several SSA problems [4]. One of these problems is failure to share the required information and observations between team members and even if this information has been shared it might be interpreted in different ways ending with different conclusions. Further, a more heterogeneous team and different reservoirs of knowledge about a specific topic, is more likely to generate different mental models. The mental models and different conclusions team members bring may have a significant impact on the projections team members need to make.

## 5. Conclusions

Although, SSA has been comprehensively investigated in other fields, this scoping review showed a clear gap in knowledge within the context of hospital emergency care. There is no standardized technique on how SSA score should be assessed and calculated. In order to advance the field of SSA in the medical emergency context, researchers might need first to ground their researching efforts to identify techniques to assess SSA and explore its impacts on team performance.

## Data Availability

The data presented are included in this study; additional data may be provided by the corresponding author on request.
